# Functional dissection of *Odorant binding protein* genes in *Drosophila melanogaster*

**DOI:** 10.1111/j.1601-183X.2011.00704.x

**Published:** 2011-06-14

**Authors:** S Swarup, T I Williams, R R H Anholt

**Affiliations:** 1 Department of Genetics, North Carolina State UniversityRaleigh, NC, USA; 2 W. M. Keck Center for Behavioral Biology, North Carolina State UniversityRaleigh, NC, USA; 3 Department of Chemistry, North Carolina State UniversityRaleigh, NC, USA; 4 Department of Biology, North Carolina State UniversityRaleigh, NC, USA

**Keywords:** Behavioral genetics, chemoreception, olfaction, proteomics, RNAi

## Abstract

Most organisms rely on olfaction for survival and reproduction. The olfactory system of *Drosophila melanogaster* is one of the best characterized chemosensory systems and serves as a prototype for understanding insect olfaction. Olfaction in Drosophila is mediated by multigene families of odorant receptors and odorant binding proteins (OBPs). Although molecular response profiles of odorant receptors have been well documented, the contributions of OBPs to olfactory behavior remain largely unknown. Here, we used RNAi-mediated suppression of *Obp* gene expression and measurements of behavioral responses to 16 ecologically relevant odorants to systematically dissect the functions of 17 OBPs. We quantified the effectiveness of RNAi-mediated suppression by quantitative real-time polymerase chain reaction and used a proteomic liquid chromatography and tandem mass spectrometry procedure to show target-specific suppression of OBPs expressed in the antennae. Flies in which expression of a specific OBP is suppressed often show altered behavioral responses to more than one, but not all, odorants, in a sex-dependent manner. Similarly, responses to a specific odorant are frequently affected by suppression of expression of multiple, but not all, OBPs. These results show that OBPs are essential for mediating olfactory behavioral responses and suggest that OBP-dependent odorant recognition is combinatorial.

*Drosophila melanogaster* provides an excellent model system for studies on olfactory behavior, as its olfactory system has been well characterized (Hallem *et al.* 2004; Su *et al.* 2009; Vosshall *et al.* 2000; Wang *et al.* 2003) and flies are readily amenable to genetic, neuroanatomical, electrophysiological and behavioral manipulations. Furthermore, virtually unlimited numbers of individuals of the same genotype can be grown under controlled environmental conditions.

Olfaction is mediated by olfactory sensory neurons (OSNs) in sensilla of the third antennal segments and the maxillary palps. In basiconic sensilla, each OSN expresses one or sometimes two unique odorant receptors from a repertoire of 62 *odorant receptor* (*Or*) genes (Robertson *et al.* 2003; Su *et al.* 2009). Each unique receptor dimerizes with a common odorant receptor (Larsson *et al.* 2004), Or83b, and activation of this complex by odorants results in the opening of a cation channel which depolarizes the OSN (Sato *et al.* 2008; Wicher *et al.* 2008). Combinatorial activation of odorant receptors generates a spatial and temporal pattern of neural activity among the population of OSNs that is relayed to the antennal lobes, where it is transformed into an activation pattern of glomeruli (Ng *et al.* 2002; Wang *et al.* 2003). Information contained within these glomerular activation maps is transmitted via projection neurons to the mushroom bodies and lateral horn of the protocerebrum in the brain (Jefferis *et al.* 2007; Lin *et al.* 2007; Luo *et al.* 2010; Wong *et al.* 2002), where olfactory information is interpreted and coupled to an appropriate behavioral output that can be modulated by prior experience (Davis 2005). In addition to the Or receptors, OSNs in coeloconic sensilla express members of a family of ionotropic (Ir) odorant receptors (Benton *et al.* 2009), which have been implicated in sensing alcohol, water vapor, amines (Yao *et al.* 2005) and acid (Ai *et al.* 2010).

The *D. melanogaster* genome also encodes a large family of *Odorant binding protein* (*Obp*) genes (Galindo & Smith 2001; Hekmat-Scafe *et al.* 2002; Graham & Davies 2002). Odorant binding proteins (OBPs) are secreted in the perilymph by support cells in the sensilla. Support for a role of OBPs in odorant recognition comes from the finding that the Lush OBP is an essential mediator of the response to the courtship pheromone 11-*cis*-vaccenylacetate (Laughlin *et al.* 2008; Xu *et al.* 2005). In addition, a 4 bp insertion in the *Obp57e* gene in *D. sechellia* results in loss of avoidance of hexanoic acid and octanoic acid produced by its host plant, *Morinda citrifolia*, and supports host plant specialization of this species (Matsuo *et al.* 2007). Furthermore, association analyses in a population of inbred wild-derived lines showed associations of polymorphisms in the *Obp99a–d* group with phenotypic variation in responses to benzaldehyde (Wang *et al.* 2007) and acetophenone (Wang *et al.* 2010). However, a systematic analysis of the roles of OBPs in mediating behavioral responses to odorants has not yet been performed. Here, we used targeted RNAi-mediated suppression of *Obp* expression and quantified behavioral responses to a battery of odorants to characterize the behavioral response profiles of OBPs.

## Materials and methods

### Drosophila stocks

Seventeen lines that express RNAi corresponding to *Obp* transcripts under *UAS* promoters inserted in the neutral phiC31 integration site along with the progenitor control line (*y,w[1118];P{attP,y[+],w[3‘]}*) were obtained from the Vienna Drosophila RNAi Center (http://www.vdrc.at). Each of these lines and the progenitor control was crossed to a ubiquitous *tubulin-GAL4* driver line (*y*^1^ w^*^; *P*{*tubP-GAL4*}*LL7/TM3, Sb*^*1*^) to suppress the expression of the target *Obp* gene. F1 offspring was used for both molecular and behavioral experiments. The lines were reared in large mass cultures on cornmeal/molasses/agar medium at 25°C and a 12 h/12 h light/dark cycle (lights on at 0600 h; lights off at 1800 h).

### Assessment of Obp gene expression levels

The efficiency of RNAi-mediated suppression of individual *Obp* genes in *tubulin-GAL4/UAS-ObpRNAi* F1s was assessed by quantitative real-time polymerase chain reaction (qRT-PCR) using an SYBR green detection method according to the protocol from Applied Biosystems (Foster City, CA, USA) with glyceraldehyde-3-phosphate dehydrogenase as the internal standard. Independent triplicates of total RNA were extracted from males and females separately using Trizol reagent (Invitrogen, Inc, Carlsbad, CA, USA). Complementary DNA was generated from 80 to 100 ng of total RNA by reverse transcription and each extract was analyzed in duplicate. Transcript-specific primers were designed to amplify 100–150 bp fragments. Negative controls without reverse transcriptase were run to exclude genomic contamination. Statistically significant differences in *Obp* gene expression levels between *tubulin-GAL4/UAS-RNAi* F1s and *tubulin-GAL4/progenitor* F1s were determined by two-tailed Student's *t* tests.

### Relative quantification of protein levels in antennae

Antennal extracts (500/sex/line) were collected from *tubulin-GAL4/ UAS-RNAi-Obp28a, tubulin-GAL4/UAS-RNAi-Obp83a* and *tubulin-GAL4/progenitor* lines and processed for proteomics analysis, essentially as described previously (Anholt & Williams 2010) with modifications as indicated below. Tryptic fragments of antennal proteins were detected by reversed-phase high-performance liquid chromatography and tandem mass spectrometry (nanoLC/MS/MS) using an Eksigent nano-LC-2D system coupled to an LTQ-Orbitrap mass spectrometer (ThermoScientific, Inc, Waltham, MA, USA). LC solvents used were mobile phase A [H_2_O/acetonitrile/HCOOH (90/10/0.2% by volume)] and mobile phase B [acetonitrile/H_2_O/HCOOH (90/10/0.2% by volume)]. The MS method consisted of seven events: a precursor scan followed by six data-dependent tandem MS scans of the first to sixth most abundant peaks in the ion trap. A high resolving power precursor scan of the eluted peptides was obtained using the LTQ-Orbitrap (60 000 resolution) with the six most abundant ions selected for MS/MS in the ion trap through dynamic exclusion. This allowed for coverage of low- and high-abundance peptides/proteins. The instrument was externally calibrated according to the manufacturer's protocol. The nanoLC/MS/MS data files were processed using MASCOT (Matrix Science, Inc, Boston, MA, USA) for protein identifications. Batch searching of nanoLC/MS/MS data was performed using the *D. melanogaster* protein database downloaded from the InterPro website (http://www.ebi.ac.uk/interpro). A Perl script version 5.8.8.820 was used to create a reverse sequence database for the *D. melanogaster* protein database (target). Target and reverse sequences were combined into one FASTA file for MASCOT batch searching of nanoLC/MS/MS data to account for the false discovery rate (FDR). A protein FDR of <1% is considered adequate and was used in determining reliable protein identifications. ProteoIQ software version 2.1.12 (BioInquire, LLC 2010, Bogart, GA, USA) was used for data normalization and relative quantification. Data were normalized by performing total spectral count normalization by standardizing to the total spectral counts for all proteins identified in each biological group and replicate. The normalization factors are calculated such that the total spectral counts for all proteins in each replicate and biological sample are equal. The normalization factors are then applied to the spectral counts for each protein. Significant differences in spectral counts between OBPs in control samples and in lines in which *Obp28a* or *Obp83a* were targeted by RNAi were assessed by Dunnett's test.

### Behavioral assay

Odorants were purchased from Sigma-Aldrich (St. Louis, MO, USA) and were of the highest purity available. Olfactory behavior of single-sex groups of 50 flies/replicate and three replicates/sex was measured for each line between 1400 and 1600 h, using a modification of the well-established ‘dipstick assay’ (Anholt *et al.* 1996). Flies that are between 4 and 7 days old were collected a day prior to the assay and food deprived for about 2 h in a 50 ml conical tube containing a cotton swab tip (referred to as ‘odor tube’). The measurement is initiated by depositing 0.1 ml of odorant solution on the cotton swab tip in the odor tube. The odor tube is then connected to a collection tube and flies are given 2 min to partition between the tubes. At the end of the assay, a response index (RI) is calculated as follows:


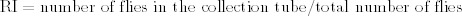


RI = 1 indicates the highest aversive response to the odorant, RI = 0.5 shows unresponsiveness to the odorant and RI = 0 indicates maximal attraction. Three replicates are run at the same time of day for three consecutive days to average environmental variation. All *ObpRNAi* lines were measured contemporaneously for each odorant along with a control (i.e. flies that carry the *tubulin-GAL4* driver without a UAS transgene in the same genetic background). Data were analyzed using a three-way analysis of variance (anova) model, *Y* = *μ* + *L* + *S* + *O* + *L*×*S* + *L*×*O* + *S*×*O* + *L*×*S*×*O* + *E*, where *μ* is the overall mean, *L* the random effect of *ObpRNAi* lines, *S* the fixed effect of sex, *O* the random effect of odorant and *E* the environmental variance. The data were further analyzed by odorant and sex using a reduced one-way anova model, *Y* = *μ* + *L*+ E, where *μ* is the overall mean, *L* the random effect of *tubulin-GAL4/UAS-ObpRNAi* lines and *E* the environmental variance. Analyses of variance and tests of significance were calculated using the Proc GLM procedure in SAS (SAS Institute, Cary, NC, USA).

## Results

### Specific RNAi-mediated suppression of Obp gene expression

To systematically dissect the functions of members of the *Obp* gene family in olfactory behavior, we knocked down expression of individual OBPs by crossing a *tubulin-GAL4* driver line to *UAS-RNAi* lines from the Vienna Drosophila RNAi Center. The *UAS-RNAi* constructs are inserted in a defined phiC31 integration site on the second chromosome in an isogenic background. The phiC31 integration site allows efficient GAL4-mediated expression and insertion of UAS constructs in this site does not give rise to positional effects (Groth *et al.* 2004). As a promoter-GAL4 construct specific for antennal support cells is not available and *Obp* genes are expressed in multiple chemosensory organs (Galindo & Smith 2001) that can affect olfactory behavior, we chose an unbiased experimental design in which we suppressed expression of *Obp* genes with a universal *tubulin-GAL4* driver. We selected 17 *UAS-RNAi* lines targeting *Obp* genes. These lines provided viable offspring when crossed to the *tubulin-GAL4* driver line with normal morphology, development time and fertility, except the *tubulin-GAL4/UAS-Obp56dRNAi* offspring and males of the *tubulin-GAL4/UAS-Obp58bRNAi* line, for which we could not obtain behavioral measurements due to poor viability.

**Figure 1:**
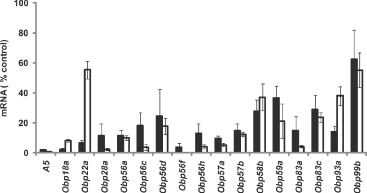
RNAi-mediated knockdown of *Obp* gene expression. Significant suppression of mRNA levels of target genes was assessed by two-tailed Student's *t* test and was significant at *P* < 0.05 for all cases, except *Obp99b* (*P* < 0.06). Solid bars represent males (*n* = 15) and open bars represent females (*n* = 15). The error bars indicate standard error of the mean.

Messenger RNA (mRNA) levels assessed by quantitative RT-PCR on whole flies were significantly reduced in the *tubulin-GAL4/UAS-RNAi* F1s compared with the corresponding control (Fig. 1). Average reduction in transcript expression across all the lines was 83% compared with control and ranged from greater than 90% (e.g. *A5*, *Obp56a*, *Obp56f*, *Obp57a*) to 40–50% (*Obp99b*).

**Figure 2:**
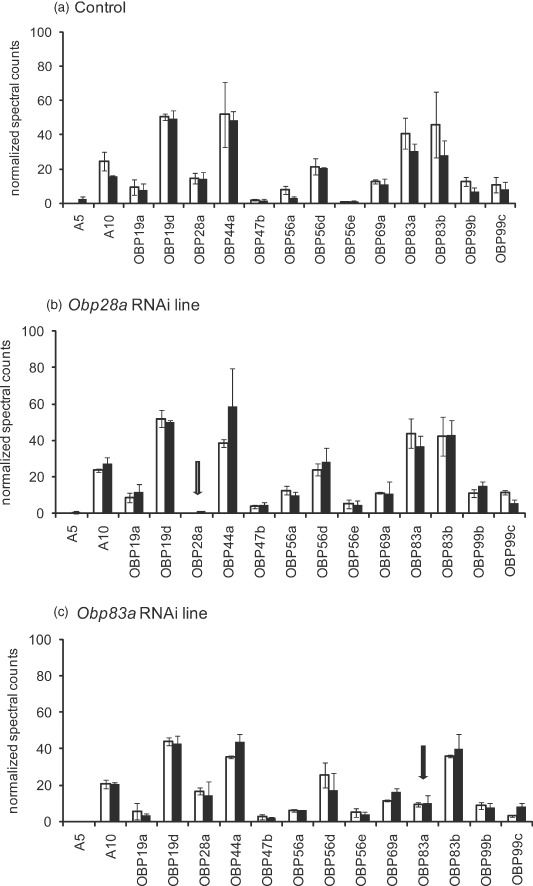
Identification and relative quantification of OBPs in antennal extracts. (a) LC/MS/MS detects 17 OBPs in offspring from the progenitor control line (*y,w[1118]; P{attP,y[+],w[3‘]}*) crossed to the *tubulin-GAL4* driver line. (b) Expression of OBP28a is specifically reduced in the *Obp28a* RNAi line (Dunnet's test, *P* < 0.05; arrow). (c) Expression of OBP83a is specifically reduced in the *Obp83a* RNAi line (Dunnet's test, *P* < 0.05; arrow). Reduction in protein levels is proportional to reduction in corresponding RNA levels (Fig. 1). Solid bars represent males and open bars represent females. The error bars indicate the standard error of the mean.

To assess to what extent reduction in transcript levels correlated with reduction in protein levels and to examine whether suppression of expression of the target *Obp* gene elicited compensatory changes in expression levels of other OBPs, we used a previously developed nanoLC/MS/MS procedure with femtomole detection sensitivity (Anholt & Williams 2010) to detect soluble proteins from the antennae of *tubulin-GAL4/UAS-Obp28aRNAi*, *tubulin-GAL4/UAS-Obp83aRNAi* and control flies. We focused on these lines because high levels of OBP28a (also known as PBPRP5) and OBP83a (also known as PBPRP3) are readily detectable in *Drosophila* antennae (Anholt & Williams 2010; Pikielny *et al.* 1994). We detected peptides derived from 237 soluble proteins (FDR < 0.01) in the antennal extracts (Table S1), including 18 OBPs (including antennal proteins A5 and A10; Fig. 2). Other members of the OBP family were not detected, because they may be present in minute amounts, are not effectively released from the antenna [e.g. detection of Lush expressed in trichoid sensilla is sporadic (Anholt & Williams 2010)] or expressed in other chemosensory tissues, such as the maxillary palps (Galindo & Smith 2001), proboscis (Cameron *et al.* 2010; Galindo & Smith 2001), tarsi (Galindo & Smith 2001; Matsuo *et al.* 2007) or wing margins.

Relative quantification of protein levels showed that reduction in the levels of OBP28a (Fig. 2b) and OBP83a (Fig. 2c) compared with control (Fig. 2a) paralleled the reduction in their respective transcript levels (Fig. 1) and was specific for each target gene without significantly affecting the expression of other OBPs or non-OBP proteins (Table S1).

### Behavioral analyses of lines with reduced expression of specific OBPs

We determined the behavioral response profiles to a panel of 16 naturally occurring and ecologically relevant odorants (Hallem & Carlson 2006; Keller & Vosshall 2007) for each *tubulin-GAL4*/*UAS-ObpRNAi* line and the control. Odorants encompassed different functional classes, including aldehydes (propanal, benzaldehyde, E2-hexenal, citral), ketones (2-heptanone, acetophenone), aromatics (phenyl ethyl alcohol, benzaldehyde, acetophenone, methyl salicylate), alcohols (1-hexanol, geraniol), esters (ethyl acetate, isoamyl acetate), terpenes (the enantiomers l-carvone and d-carvone), and pyrazines (2-methylpyrazine, 2-ethylpyrazine). Pilot dose–response studies established 1% (v/v) as a maximally discriminating odorant concentration (Fig. S1), which provides overall good signal-to-noise resolution while remaining below the maximum aversive response of RI = 1. Response indices were measured for each odorant for each *UAS-ObpRNAi* line and compared with contemporaneously measured response indices of the control. We analyzed the data with three-way anova to assess statistically significant differences and found a significant line × odorant effect for all *tubulin-GAL4/UAS- ObpRNAi* lines, except *Obp56h* (which showed a significant line × sex effect; Table S2). Subsequently, we used a reduced anova model to identify odorant-specific and sex-specific effects for each *tubulin-GAL4/ UAS-ObpRNAi* line.

**Figure 3:**
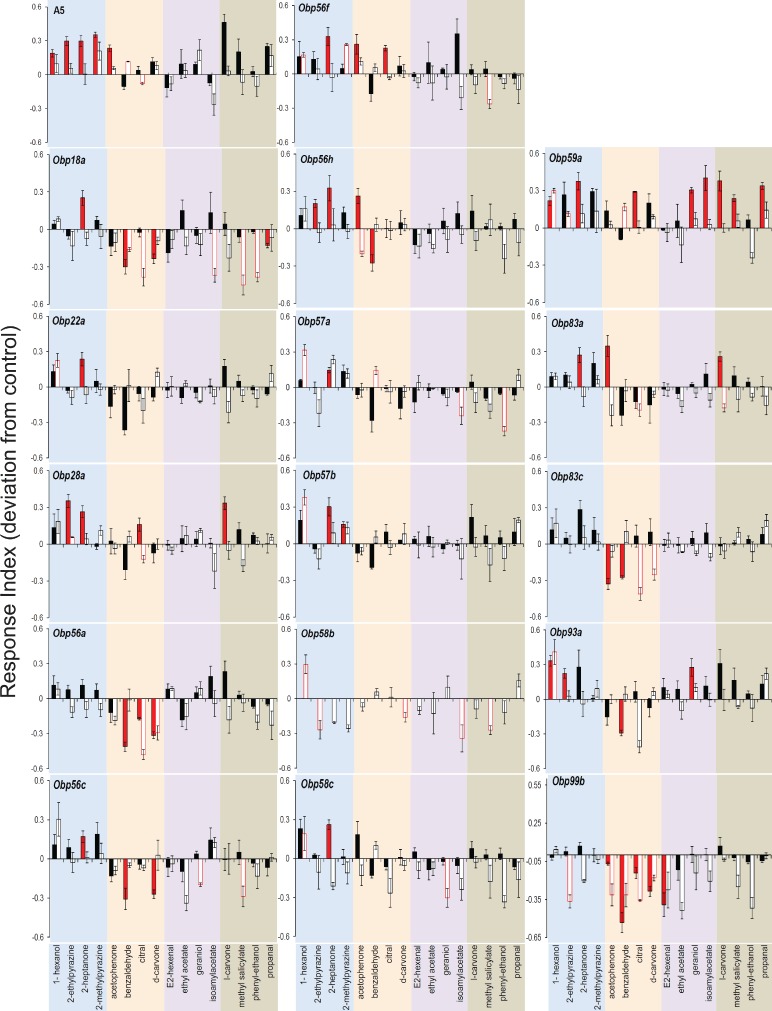
Effects of RNAi-mediated suppression of *Obp* expression on behavioral responses to odorants. Each panel represents an RNAi-targeted *Obp* transcript. Odorants are indicated along the *x*-axes of the panels. RI is indicated along the *y*-axes. Statistically significant differences between behavioral responses of RNAi lines and controls were determined by anova and are presented as deviations from the mean RI of the control line (RI [knockdown] − RI [control]). Solid bars represent males and open bars represent females. Red color indicates statistically significant differences from control (*P* < 0.05). The bars indicate averages and standard errors of three measurements with 50 flies each.

Disruption of expression of individual OBPs results in altered behavioral responses to multiple, but not all, odorants, and affects males and females differently (Fig. 3). For example, disruption of expression of OBP28a shows altered behavioral responses to 2-heptanone and l-carvone in males and to 2-ethylpyrazine and citral in both sexes. Sexual dimorphism is also evident for OBP83a, where reduction in expression results in altered responses to l-carvone in both sexes, to 2-heptanone and acetophenone in males and to citral in females (Fig. 3). Some OBPs show narrowly tuned behavioral response profiles (e.g. *Obp22a* and *Obp57b*), whereas others have broad response patterns (e.g. *Obp59a* and *Obp99b*), as inferred from targeted RNAi-mediated suppression with this limited panel of odorants. To further assess the reproducibility of our measurements, we selected RNAi lines targeting the abundantly expressed OBPs, *Obp28a* and *Obp83a* (Fig. 2), for further testing with contemporaneous controls. We conducted five additional behavioral measurements for those odorants (citral and l-carvone, respectively) that showed significant differences from the control in the initial screen, for sexes separately, at four different odorant concentrations and replicated the data from our initial screen at 1% (v/v) odorant concentration (Fig. S2).

**Figure 4:**
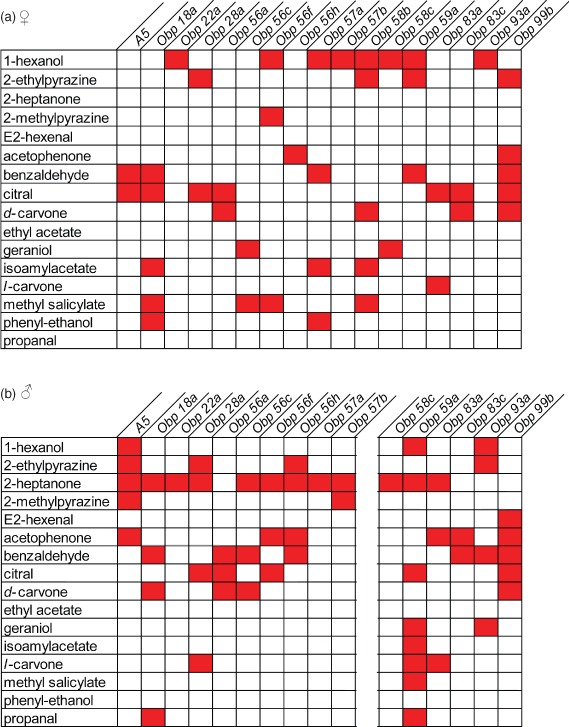
Combinatorial response profiles of OBPs in *Drosophila melanogaster* females (a) and males (b) inferred from RNAi-mediated suppression of *Obp* transcripts. Significant differences from control (*P* < 0.05) are indicated in red. The diagram is based on Fig. 3.

It is noteworthy that structurally similar odorants, such as benzaldehyde and acetophenone, and 2-methylpyrazine and 2-ethylpyrazine are associated with distinct, albeit overlapping, behavioral profiles among the 17 *tubulin-GAL4/ UAS-ObpRNAi* lines. Odorants differ widely in their dependence on expression of different OBPs to elicit behavioral responses. For example, olfactory responses to propanal are only disrupted in males in which the expression of *Obp18a* or *Obp59a* is suppressed, whereas responses to many other odorants are altered in more lines with reduced OBP expression, with different patterns between males and females (Figs 3 and 4). It is possible that differential efficacies of RNAi-mediated knockdown in males and females may account for some of the observed sex differences (e.g. *Obp22a* and *Obp93a*, Fig. 1). However, in some instances greater inhibition of *Obp* expression in one sex resulted in a smaller effect on olfactory behavior than in its counterpart where reduction in expression of the same *Obp* was less affected (e.g. *Obp28a*, Fig. 1) and in cases where reduction in *Obp* transcript was similar in males and females, sexually dimorphic behavioral effects were still observed (e.g. *Obp56a* and *Obp83c*, Figs 1 and 3).

## Discussion

We used targeted RNAi-mediated suppression of *Obp* expression to quantify the behavioral response profiles of OBPs to a battery of odorants. The extent of RNAi-mediated knockdown of *Obp* gene expression varied among the *tubulin-GAL4/UAS-RNAiObp* lines and there was no linear relationship between suppression of *Obp* gene expression and behavioral effects. It should be noted that we measured odorant responses at a single concentration and that the response profiles shown in Figs 3 and 4 may shift at different odorant concentrations or with different *Obp* expression levels (Zhou *et al.* 2009). Only five of the OBPs examined are readily detectable in antennal extracts (OBP28a, OBP56a, OBP56d, OBP83a and OBP99b; Fig. 2). This can be because of suboptimal release of OBPs from specific antennal sensilla or limitations of detection by the LC/MS/MS procedure. Alternatively, behavioral responses to odorants could result from integration of input from multiple chemosensory organs, e.g. the maxillary palps. Although we have examined behavioral response profiles of only about one third of the *Obp* family with a limited panel of odorants, we believe that the scope of our study provides a representative illustration of the role of OBPs in olfactory behavior.

### Combinatorial recognition of odorants inferred from targeted RNAi-mediated inhibition of Obp expression

RNAi-mediated reduction in expression of a single *Obp* gene results in altered behavioral responses to multiple, but not all, odorants, and responses to a given odorant are affected by reduced expression of multiple, but not all, *Obp* genes (Figs 3 and 4). This observation suggests, at least indirectly, that interactions between OBPs and odorants are combinatorial. Combinatorial recognition of odorants by OBPs and odorant receptors implies that perception of odor quality depends on the activation pattern that arises across multiple glomeruli in the antennal lobe (Ng *et al.* 2002; Wang *et al.* 2003). Alterations in this glomerular activation map by attenuating a single input component are predicted to result in altered perception of odor quality that can give rise to either enhanced or reduced behavioral responses. This prediction is in line with our observation that reduction in expression of a single *Obp* gene sometimes results not only in decreased but also in increased responses to specific odorants (Fig. 3). Comprehensive binding studies with odorants could further corroborate the extent and overlap of molecular response profiles of OBPs.

### Sex-specific effects of RNAi-mediated reduction in Obp expression

Analysis of whole-genome transcript profiles of 40 inbred wild-derived *D. melanogaster* lines showed that the transcriptome is highly organized with 241 covariant modules of genetically variable transcripts. A vast proportion of the transcriptome showed sex-biased expression with extensive male–female antagonism (Ayroles *et al.* 2009). Expression of chemoreceptor genes, notably *Obp*s, showed especially extensive sexual dimorphism, even among *Obp* genes located within the same chromosomal cluster (Zhou *et al.* 2009). Moreover, sexually dimorphic differences in expression levels of *Obp* transcripts have been documented in response to different environmental, physiological and social conditions (Zhou *et al.* 2009). These observations indicated that males and females utilize the chemoreceptor repertoire differently to perceive their chemosensory environment (Zhou *et al.* 2009). Because mating can affect *Obp* expression (McGraw *et al.* 2004; Zhou *et al.* 2009), it is possible that behavioral response profiles of the *RNaiObp* lines would show differences in virgin flies. However, this does not affect our conclusions, as we compare only mated flies throughout this study. In addition, sexual dimorphism in responses to a single odorant, benzaldehyde, in *P*-element insertion lines (Anholt *et al.* 1996; Sambandan *et al.* 2006) and in chromosome substitution lines (Mackay *et al.* 1996) has been reported previously. It is, therefore, not surprising that we found that the effects of suppression of *Obp* expression on behavioral responses to odorants were sexually dimorphic. It should be noted that combinatorial recognition of odorants would accentuate sexual dimorphism of behavioral responses in our *RNAi-Obp* lines, because reduction of a single sex-biased component within an ensemble of responsive OBPs could result in divergent changes in overall activation patterns, and, hence, odor quality perception, between the sexes. Thus, the functional consequences of knockdown of each *Obp* transcript are dependent on the context of sex-specific expression of the entire *Obp* repertoire.

### Relationships between OBPs and odorant receptors in odorant detection

**Figure 5:**
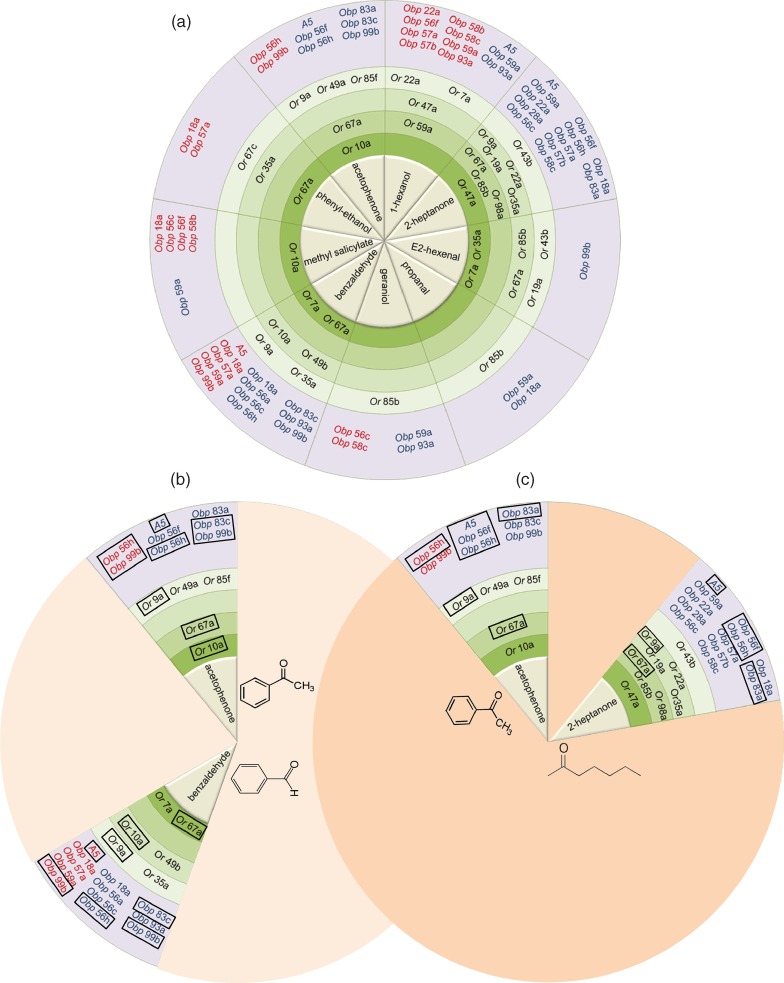
Relationships between electrophysiological response profiles of odorant receptors and behavioral response profiles of OBPs. (a) The innermost circle represents nine odorants and odorant receptors responding to each odorant are indicated from the center to the periphery in concentric green circles in descending order of responsiveness of >200, >150, >100 and >50 spikes/seconds, respectively (Hallem & Carlson 2006). The outer purple circle indicates *Obp*s of which reduced expression affects responses to these odorants in males (blue) and females (red). (b) This panel highlights relationships between electrophysiological response profiles of odorant receptors and behavioral response profiles of OBPs to the structurally similar odorants, acetophenone and benzaldehyde. (c) This panel highlights relationships between electrophysiological response profiles of odorant receptors and behavioral response profiles of OBPs to odorants with a similar functional group, in this example, the ketones acetophenone and 2-heptanone. In panels (b) and (c), odorant receptors and OBPs common for both odorants are outlined in boxes.

Other than the previously reported interaction between the pheromone binding protein Lush and Or67d (Laughlin *et al.* 2008; Xu *et al.* 2005), there is currently no evidence for precise functional relationships between OBPs and odorant receptors. Although there is no simple linear relationship between behavioral response profiles of OBPs and molecular response profiles of odorant receptors (Hallem *et al.* 2004), features of functional organization emerge between behavioral response profiles of OBPs and electrophysiological response profiles of odorant receptors (Hallem & Carlson 2006). For example, structurally similar odorants such as benzaldehyde and acetophenone activate common odorant receptors, Or9a, Or10a and Or67a, and behavioral responses to both odorants are suppressed by inhibition of *Obp56h*, *Obp99b*, *Obp83c* and *A5* (Fig. 5a,b). Similarly, common odorant receptors and *Obps* can respond to odorants carrying the same functional groups. For example, acetophenone and 2-heptanone are ketones that both activate Or9a and Or67a; behavioral responses to both these odorants are affected by reduced expression of *A5*, *Obp56f*, *Obp56h* and *Obp83a* (Fig. 5a,c).

We also note that Or85b responds to propanal and 2-heptanone, and behavioral responses to both these odorants are suppressed by inhibition of the expression of *Obp18a* and *Obp59a* in males. Or10a is activated by methylsalicylate and acetophenone; responses to these odorants are affected by suppression of expression of *Obp56f* in females for methylsalicylate and males for acetophenone (Fig. 5a).

Furthermore, we noted that odorant receptors that are activated by odorants of the same functional class, but expressed in different sensilla (Hallem *et al.* 2004), may interact with common OBPs. For example, Or47a, expressed in ab5B neurons, and Or43b, expressed in ab8A neurons, are both activated by 2-heptanone, and Or10a, expressed in ab1D neurons, is activated by acetophenone. Acetophenone and 2-heptanone are ketones and the behavioral responses to both are affected by suppression of *A5*, *Obp56h*, *Obp56f* and *Obp83a* (Figs 3, 4 and 5a,c). Similarly, suppression of *Obp59a* affects the behavioral responses to both propanal, which activates Or85b, expressed in ab3B neurons, and benzaldehyde, which activates Or10a, expressed in ab1D neurons (Fig. 5a).

A similar pattern is observed for structurally similar odorants. For example, Or10a, expressed in ab1D neurons, is activated by acetophenone, and Or7a, expressed in ab4A neurons, is activated by benzaldehyde (Hallem *et al.* 2004). The behavioral responses to both these odorants are affected by suppression of *A5*, *Obp56h*, *Obp99b* and *Obp83c* (Fig. 5a,b).

Finally, behavioral responses to odorants with different functional groups that activate the same odorant receptor can also be affected by suppressed expression of common OBPs. For example, reduced expression of *Obp59a* altered behavioral responses to propanal and geraniol, both of which activate Or85b, expressed in the ab3B neuron (Hallem *et al.* 2004). Thus, although further studies will be required to compare the distribution and coexpression of OBPs and odorant receptors across antennal sensilla, complex functional mosaics of combinatorial recognition patterns are likely to characterize the relationships between OBPs and odorant receptors.

Combinatorial activation of OBPs by general odorants suggested by our data contrasts with previously documented activation of OBPs by pheromones. Binding of the *Bombyx mori* pheromone bombykol proceeds via a specific pheromone binding protein (Grosse-Wilde *et al.* 2006; Wojtasek & Leal 1999) and a defined odorant receptor, BmOR-1 (Grosse-Wilde *et al.* 2006; Sakurai *et al.* 2004; Wojtasek & Leal 1999). In *D. melanogaster*, perception of 11-*cis*-vaccenylacetate is mediated via a single OBP, Lush, interacting with the Or67d receptor, in specialized T1 trichoid sensilla (Kurtovic *et al.* 2007; Laughlin *et al.* 2008). This dichotomy between strategies for pheromone recognition, which requires the identification of specific compounds with high sensitivity, and general odorant discrimination, which requires the assessment of myriads of odorants, is reminiscent of the organization of the mammalian olfactory system. Here, combinatorial activation of odorant receptors mediates olfactory discrimination in the main olfactory system (Malnic *et al.* 1999), whereas V1R pheromone receptors in the vomeronasal organ have highly specific molecular response profiles (Leinders-Zufall *et al.* 2000).

Mammalian olfactory systems have a vastly larger repertoire of odorant receptor genes [1296 odorant receptor genes in the mouse (Zhang & Firestein 2002)] but only one or few OBPs, which bind odorants with low specificity and are likely to serve mainly a transport function (Bianchet *et al.* 1996; Hajjar *et al.* 2006). Our results indicate that OBPs in *Drosophila* play an essential role in mediating olfactory behavior. In contrast to vertebrates, in insects the external soluble milieu around odorant receptors is discontinuous and sequestered in individually segregated sensilla. It is tempting to speculate that here dual chemosensory recognition, in which combinatorial activation of OBPs precedes combinatorial activation of odorant receptors, could provide an alternate mechanism to the large expansion of odorant receptors found in mammals for increasing olfactory discrimination power.

## Supporting Information

Figure S1 Dose responses for benzaldehyde, 1-hexanol andacetophenone for males and females, separately, measured in theprogenitor control line crossed to the *tubulin-GAL4* driver from the Vienna Drosophila RNAi Center.

Figure S2 Dose responses for RNAi lines *Obp28a*and *Obp83a* crossed to *tubulin-GAL4* driver for citraland L-carvone, respectively, for males and females, separately,measured contemporaneously with progenitor control line crossed tothe *tubulin-GAL4* driver.

Table S1 Identification of proteins by LC/MS/MS inantennal extracts expressing RNAi targeted against *Obp28a*,*Obp83a* and the progenitor control.

Table S2 Three-way ANOVA of behavioral responses of*tubulin-GAL4/UAS-ObpRNAi* lines.

Table S3 Raw data for results in Fig. 3 as deviations ofresponse indices of *tubulin-GAL4/UAS-ObpRNAi* lines from control values.

As a service to our authors and readers, this journal provides supporting information supplied by the authors. Such materials are peer-reviewed and may be re-organized for online delivery, but are not copy-edited or typeset. Technical support issues arising from supporting information (other than missing files) should be addressed to the authors.
