# Supplementary material for: Functional dissection of Odorant binding protein genes in Drosophila melanogaster
Source: Genes Brain Behav. 2011 Jun 14;10(6):648–57. doi: 10.1111/j.1601-183X.2011.00704.x (PMC3150612; doi:10.1111/j.1601-183X.2011.00704.x)

Fig. S1: Dose responses for benzaldehyde, 1-hexanol and acetophenone for males and females separately measured in progenitor control line crossed to tubulin-GAL4 driver from Vienna Drosophila RNAi Center.

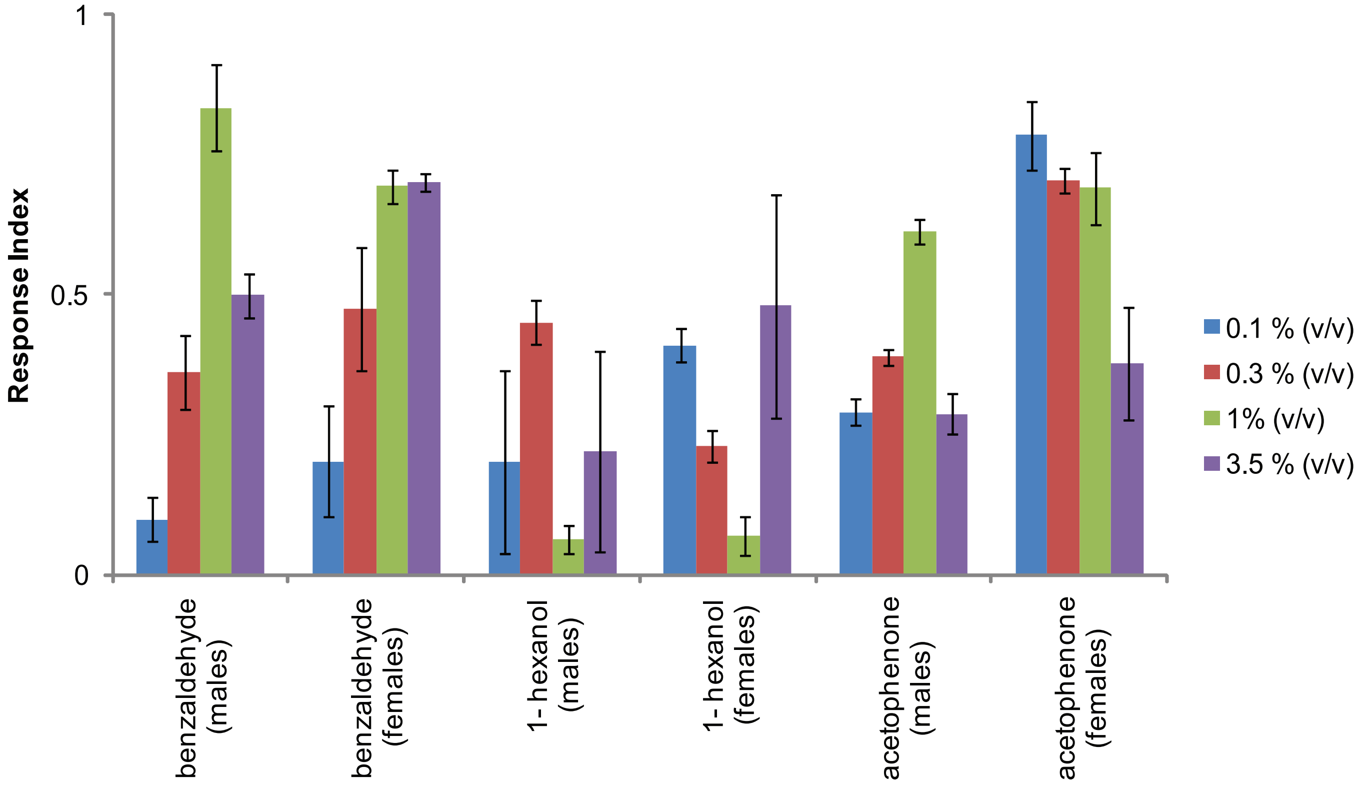

Supplement: Figure S1 — Dose responses for benzaldehyde, 1-hexanol andacetophenone for males and females, separately, measured in theprogenitor control line crossed to the tubulin-GAL4 driver from the Vienna Drosophila RNAi Center. [file gbb0010-0648-SD1.pdf]
