# Supplementary material for: Functional dissection of Odorant binding protein genes in Drosophila melanogaster
Source: Genes Brain Behav. 2011 Jun 14;10(6):648–57. doi: 10.1111/j.1601-183X.2011.00704.x (PMC3150612; doi:10.1111/j.1601-183X.2011.00704.x)

Figure S2: Dose responses for RNAi lines targeting *Obp28a* and *Obp83a* crossed to the *tubulin-GAL4* driver for citral and l-carvone, respectively,for males and females separately, measured contemporaneously with the progenitor control line crossed to the *tubulin-GAL4* driver. Boxed graphs indicate the concentration of odorant used to screen responses to all odorants among all lines (* p< 0.05, ** p< 0.01, *** p< 0.001, two-tailed Student's t-test). Open bars, controls; closed bars, *Obp*RNAi lines.

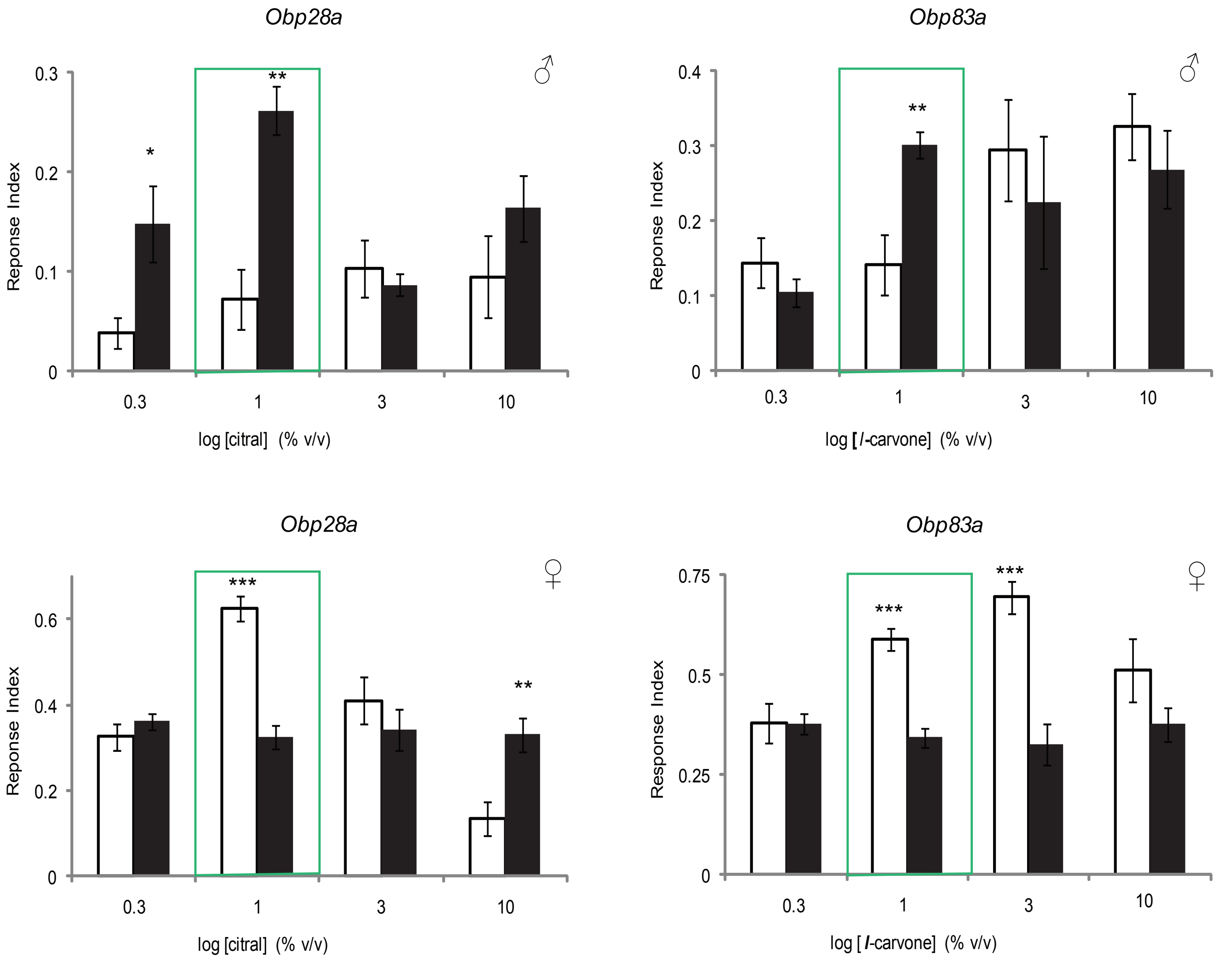

Supplement: Figure S2 — Dose responses for RNAi lines Obp28aand Obp83a crossed to tubulin-GAL4 driver for citraland L-carvone, respectively, for males and females, separately,measured contemporaneously with progenitor control line crossed tothe tubulin-GAL4 driver. [file gbb0010-0648-SD2.pdf]
