# Supplementary material for: Functional dissection of Odorant binding protein genes in Drosophila melanogaster
Source: Genes Brain Behav. 2011 Jun 14;10(6):648–57. doi: 10.1111/j.1601-183X.2011.00704.x (PMC3150612; doi:10.1111/j.1601-183X.2011.00704.x)
Supplement: Table S2 — Three-way ANOVA of behavioral responses oftubulin-GAL4/UAS-ObpRNAi lines. [file gbb0010-0648-SD4.doc]

**Supplementary Table S2**

**Three-way Analysis of Variance of behavioral responses of *Obp* RNAi lines**

source df^a^ Mean Square F *P*

*Obp18a*

line 1 0.4892462035 36.4278 1.6e-8

sex 1 2.7706305107 206.293 2e-28

odorant 15 0.2995450273 22.3032 1e-28

sex*odorant 15 0.0426760993 3.17754 0.0002

line*sex 1 0.2374106603 17.6769 4.9e-5

line*odorant 15 0.0305672062 2.27594 0.007

line*sex*odorant 15 0.0345592607 2.57318 0.0022

Error 128 0.01343056

*Obp* *22a*

line 1 0.0264221302 2.20372 0.1401

sex 1 4.4612447213 372.087 1e-39

odorant 15 0.2964957251 24.729 1e-30

sex*odorant 15 0.0447141863 3.72936 2e-5

line*sex 1 0.001568419 0.13081 0.7182

line*odorant 15 0.0208297599 1.73729 0.0514

Error 128 0.01198978

*Obp28a*

line*sex*odorant 15 0.0283028602 2.36058 0.005

line 1 0.0839368387 7.00081 0.0092

sex 1 3.5904735391 299.466 3e-35

odorant 15 0.2843769924 23.7187 7e-30

sex*odorant 15 0.0473481066 3.9491 8.1e-6

line*sex 1 0.0660051442 5.5052 0.0205

line*odorant 15 0.0237689716 1.98247 0.0212

line*sex*odorant 15 0.0265408425 2.21366 0.0088

Error 128 0.01198959

*Obp56a*

line 1 0.2252875304 17.919 4.4e-5

sex 1 3.3300073732 264.863 6e-33

odorant 15 0.3372585731 26.825 2e-32

sex*odorant 15 0.0426861898 3.39519 0.0001

line*sex 1 0.1068887005 8.50175 0.0042

line*odorant 15 0.0569286492 4.52801 7.8e-7

line*sex*odorant 15 0.0254381104 2.02331 0.0182

Error 128 0.01257255

*Obp56c*

line 1 0.0398313237 3.01427 0.0849

sex 1 4.1904453632 317.116 2e-36

odorant 15 0.2768909523 20.954 2e-27

sex*odorant 15 0.0584901318 4.42629 1.2e-6

line*sex 1 0.0109644815 0.82975 0.3641

line*odorant 15 0.0418444338 3.16661 0.0002

line*sex*odorant 15 0.0244505134 1.85031 0.0344

Error 128 0.01321426

*Obp56f*

line 1 0.0619769509 3.91978 0.0499

sex 1 3.0290806341 191.576 3e-27

odorant 15 0.3470954024 21.9523 2e-28

sex*odorant 15 0.0572593904 3.62141 3.1e-5

line*sex 1 0.1692036691 10.7014 0.0014

line*odorant 15 0.0275886738 1.74487 0.0501

line*sex*odorant 15 0.0290003203 1.83415 0.0365

Error 128 0.01581134

*Obp56h*

line 1 0.0025824775 0.16466 0.6856

sex 1 3.0357951421 193.563 2e-27

odorant 15 0.2887905272 18.4134 4e-25

sex*odorant 15 0.0597228706 3.80795 1.4e-5

line*sex 1 0.1676213129 10.6876 0.0014

line*odorant 15 0.0242185625 1.54418 0.0991

line*sex*odorant 15 0.0240319596 1.53228 0.103

Error 128 0.01568374

*Obp57a*

line 1 0.0484338426 4.4751 0.0363

sex 1 4.8240353254 445.722 2e-43

odorant 15 0.3223712313 29.7859 2e-34

sex*odorant 15 0.036761197 3.39659 0.0001

line*sex 1 0.0019891167 0.18379 0.6689

line*odorant 15 0.039497842 3.64945 2.7e-5

line*sex*odorant 15 0.0284111629 2.62508 0.0018

Error 128 0.01082296

*Obp57b*

line 1 0.0624822977 4.34807 0.039

sex 1 4.0058518744 278.763 6e-34

odorant 15 0.2903139823 20.2026 8e-27

sex*odorant 15 0.0475542649 3.30925 0.0001

line*sex 1 0.0225921099 1.57216 0.2122

line*odorant 15 0.035728414 2.4863 0.0031

line*sex*odorant 15 0.0158230498 1.10111 0.3619

Error 128 0.01437011

*Obp58b*

line 1 0.1794583681 12.6273 0.0006

sex 1 2.315055245 162.896 2e-22

odorant 15 0.2502964206 17.6118 3e-21

sex*odorant 15 0.0495710378 3.488 0.0001

line*sex 0 . . .

line*odorant 15 0.0456314666 3.21079 0.0003

line*sex*odorant 0 . . .

Error 96 0.01421190

*Obp58c*

line 1 0.1029161039 7.57503 0.0068

sex 1 2.5997765607 191.354 4e-27

odorant 15 0.3798663604 27.9597 3e-33

sex*odorant 15 0.0646169156 4.75606 3.1e-7

line*sex 1 0.2909382181 21.4142 8.9e-6

line*odorant 15 0.027663805 2.03617 0.0174

line*sex*odorant 15 0.0198383518 1.46018 0.1299

Error 128 0.01358622

*Obp59a*

line 1 0.8696166808 67.971 2e-13

sex 1 2.5062524374 195.894 1e-27

odorant 15 0.2953594143 23.0859 2e-29

sex*odorant 15 0.0332382997 2.59797 0.002

line*sex 1 0.3233677854 25.2751 1.6e-6

line*odorant 15 0.0358184333 2.79964 0.0009

line*sex*odorant 15 0.0203838059 1.59324 0.0842

Error 128 0.01279393

*Obp83a*

line 1 0.0049800271 0.38803 0.5344

sex 1 2.7668588632 215.584 3e-29

odorant 15 0.3050407917 23.7678 6e-30

sex*odorant 15 0.0609538937 4.74932 3.2e-7

line*sex 1 0.2385163787 18.5844 3.2e-5

line*odorant 15 0.0234472332 1.82693 0.0374

line*sex*odorant 15 0.0288800317 2.25023 0.0077

Error 128 0.01283423

*Obp83c*

line 1 0.0002390651 0.01903 0.8905

sex 1 3.9254341221 312.534 4e-36

odorant 15 0.281590565 22.4196 8e-29

sex*odorant 15 0.0729879674 5.81113 5.1e-9

line*sex 1 0.0290696766 2.31446 0.1306

line*odorant 15 0.0320108559 2.54863 0.0024

line*sex*odorant 15 0.034404363 2.73919 0.0011

Error 128 0.01256003

*Obp93a*

line 1 0.2233239054 15.7087 0.0001

sex 1 3.716308113 261.406 1e-32

odorant 15 0.2496335428 17.5593 3e-24

sex*odorant 15 0.04302231 3.0262 0.0003

line*sex 1 0.0501743496 3.52928 0.0626

line*odorant 15 0.0438383157 3.0836 0.0003

line*sex*odorant 15 0.026878878 1.89067 0.0297

Error 128 0.01421660

*Obp99b*

line 2 0.6321894574 53.4946 1e-17

sex 1 2.7314241208 231.128 2e-30

odorant 15 0.2191821742 18.5468 3e-25

sex*odorant 15 0.049888025 4.22143 2.7e-6

line*sex 1 0.2233646844 18.9007 2.8e-5

line*odorant 15 0.0441793924 3.73837 1.9e-5

line*sex*odorant 14 0.0251017942 2.12406 0.0144

Error 128 0.01181781

*A5*

line 1 0.3802838318 30.9603 1.5e-7

sex 1 3.0471236136 248.077 1e-31

odorant 15 0.3023042857 24.6117 1e-30

sex*odorant 15 0.0548499242 4.46553 1e-6

line*sex 1 0.1649723915 13.431 0.0004

line*odorant 15 0.0472856071 3.84969 1.2e-5

line*sex*odorant 15 0.0192784438 1.56953 0.0911

Error 128 0.01228296

^a^ df, degrees of freedom
